# Hepatitis E Virus Drug Development

**DOI:** 10.3390/v11060485

**Published:** 2019-05-28

**Authors:** Volker Kinast, Thomas L Burkard, Daniel Todt, Eike Steinmann

**Affiliations:** Ruhr-University Bochum, Faculty of Medicine, Department of Molecular and Medical Virology, 44801 Bochum, Germany; Volker.kinast@rub.de (V.K.); thomas.burkard@rub.de (T.L.B.); daniel.todt@rub.de (D.T.)

**Keywords:** hepatitis E virus, antivirals, ribavirin, therapy, sofosbuvir, vaccine, drug development

## Abstract

Hepatitis E virus (HEV) is an underestimated disease, leading to estimated 20 million infections and up to 70,000 deaths annually. Infections are mostly asymptomatic but can reach mortality rates up to 25% in pregnant women or become chronic in immunocompromised patients. The current therapy options are limited to the unspecific antivirals Ribavirin (RBV) and pegylated Interferon-α (pegIFN-α). RBV leads to viral clearance in only 80% of patients treated, and is, similar to pegIFN-α, contraindicated in the major risk group of pregnant women, emphasizing the importance of new therapy options. In this review, we focus on the urgent need and current efforts in HEV drug development. We provide an overview of the current status of HEV antiviral research. Furthermore, we discuss strategies for drug development and the limitations of the approaches with respect to HEV.

## 1. Introduction

**Figure 1:**
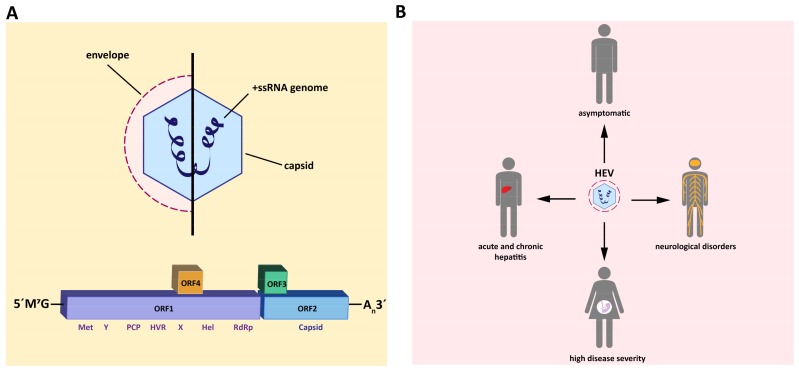
Schematic representation of hepatitis E virus (HEV) particle and the major clinical manifestations. (**A**) HEV particle and genomic organization. The HEV genome is composed of a single-stranded RNA genome of ~7.2 kb and is encapsulated in an icosahedral capsid. HEV virions can occur in both a non-enveloped and in an enveloped form. The viral RNA, which is capped with 7-methylguanosine (7mG) at the 5´noncoding region and polyadenylated at the 3′noncoding region, comprises three open reading frames (ORF). Furthermore, GT1 is believed to contain an additional ORF (ORF4). ORF1 encodes the replicase proteins, including a methyltransferase (MT), cysteine protease (Pro), helicase (Hel), and RNA polymerase (Pol), as well as three regions without a reported enzymatic function (Y, hypervariable region (HVR), and X). ORF2 encodes the capsid protein, whereas ORF3 encodes a viroporin. (**B**) Major clinical manifestations. The majority of HEV infections are asymptomatic. GT3 and GT4 infections can become chronic in immunosuppressed individuals, with high risk for developing severe complications, such as liver cirrhosis. HEV has also been reported to cause a variety of extrahepatic manifestations, like Guillain–Barré syndrome. Infections with HEV GT1 cause acute hepatitis, with high mortality rates up to 25% in pregnant women.

With approximately 20 million infected people per year, hepatitis E virus (HEV) leads to more cases of acute hepatitis than any other human hepatotropic virus, such as Hepatitis A virus (HAV), Hepatitis B virus (HBV), Hepatitis C virus (HCV) and Hepatitis D virus (HDV). HEV is a quasi-enveloped positive strand RNA virus (Figure 1A) that is classified as a member of the genus *Orthohepevirus* within the family of *Hepeviridae* [1]. The genotypes (GT) 1 and 2 of HEV are obligate human pathogens and are primarily transmitted via contaminated drinking water. Recent outbreaks of acute hepatitis linked to HEV have, amongst others, been reported in Nigeria [2], Chad [3], and Bangladesh [4]. By contrast, the zoonotic GT3 and 4, which are endemic especially in Europe and the Americas, can cause in addition to acute infections also chronic infections in immunocompromised individuals. The most common infection routes are thought to be the consumption of undercooked meat of or contact with infected animals, such as pigs, wild boars, and deer, which constitute the virus reservoir. Furthermore, the transfer of contaminated blood products is a man-made safety hazard, especially for risk groups such as immunocompromised patients [5]. Recent cases of human infections with GT7 [6] and Rat HEV [7,8] extended the spectrum of HEV GTs, which are capable of jumping over the species barrier and are able to infect humans. Acute HEV infections are self-limiting in most cases, but especially for GT1, they are linked to high mortality rates up to 25% in pregnant women [9]. It is hypothesized that immunological and hormonal changes are responsible for the high mortality [10]. HEV has also been reported to cause a variety of extrahepatic manifestations, for instance, Guillain–Barré syndrome or pancreatitis (reviewed in [11], see Figure 1B). In total, 3.3 million estimated cases of acute illness and 44,000–70,000 deaths per year make this pathogen a non-negligible health burden. However, the current therapeutic options against HEV are limited to the off-label use of the unspecific antivirals Ribavirin (RBV) and pegylated Interferon-α (pegIFN-α). The treatment algorithm for chronic infections by the European Association for the Study of the Liver (EASL) from 2018 stipulates lowering the dose of immunosuppressive drugs and, subsequently, if no viral clearance is achieved, up to two courses of RBV. If both RBV regimens fail, pegIFN-α can be administrated, but is only suited for the subset of liver-transplant recipients [12,13,14,15]. Thus, RBV is the treatment of choice but leads to viral clearance in only 80% of patients treated [16]. Similar to pegIFN-α, it is contraindicated in the major risk group of pregnant women, emphasizing the importance of new therapy options. In this review, we focus on the urgent need and current efforts in HEV drug development.

## 2. Strategies to Find Novel Therapy Options

Identification of novel therapy options can encompass several strategies. While some rely on de novo identification of compounds, another approach is to reuse already existing compounds, a process which is termed drug repurposing. Drug repurposing can generally be described as the idea of using drugs or drug candidates for another than their primary indication. The pharmacokinetic and pharmacodynamic profile, including undesirable effects, is often already elucidated in vivo in animals and humans. The current first choice of anti-HEV treatment, RBV, is an example for a repurposed drug that was originally developed for treatment of respiratory syncytial virus (RSV) in infants [17]. RBV has not only been administered for antiviral therapy against RSV but also against HCV, Influenza, viral hemorrhagic fevers [18] and HEV [19].

Recently, drug repurposing has gained increased attention in the scientific community with reviews covering either specific viruses [20] or viral diseases in general [21,22]. Surprisingly, there are only a few publications, in which this approach is used as a strategy to find an antiviral against HEV. However, given the availability of public FDA-approved compound libraries and the anticipated benefits of this approach over de novo development, publications screening for antiviral activity of already approved drugs are anticipated.

De novo drug development can rely on screens, where compound libraries are tested for their capacity to interfere with the viral life cycle. Both the target and the mode of action of the substance do not need to be identified. For structure-guided development, the target and ideally its crystal structure is already identified, which enables to specifically design antivirals.

Independent from the approach used to identify a prospect compound, the candidates have to be validated in vitro and in vivo. There are several in vitro models available, including different cell culture models as well as primary human hepatocytes and induced pluripotent stem cells. For a detailed overview, please see a recent review by Meister et al. [23]. Similarly, there are several small animal models as rabbits, rats, ferrets, and birds, which are used for the different HEV strains (reviewed in [24]). This review aims to give an overview over the current state of efforts to establish additional treatment options against HEV. Antiviral candidates are grouped according to the strategy that was used to identify them. Before the concluding remarks, vaccination is also briefly discussed in terms of its potential to replace antiviral therapy options.

## 3. Drug Repurposing

### 3.1. 2’-C-Methylcytidine

2’-C-methylcytidine (2-CMC), also known as NM107, is a nucleoside analogue (NA) originally developed against HCV. When first applied against HEV in vitro, the compound demonstrated an inhibitory effect against HEV replication (IC_50_ of 22 µM/L) [25]. Furthermore, it inhibited the replication of both a luciferase replicon based on Kernow C1 as well as the full-length virus without showing signs of resistance upon prolonged incubation [26]. The effect could be reverted by addition of cytidine triphosphate, but not guanosine triphosphate to the cells. Applying it together with RBV yielded in a moderate antagonistic effect, suggesting a convergent mechanism of inhibition. When assessed for treatment of HCV, insufficient oral bioavailability was reported. Therefore, a prodrug termed NM283 was developed [27]. However, the development was discontinued due to adverse toxic effects, which has been a problem for several 2’methyl nucleosides [28]. The toxicity correlates with the property to serve as a substrate for the mitochondrial DNA Polymerase and thus leading to the termination of mitochondrial RNAs [29]. The example of NM283 emphasizes that 2-CMC, although efficacious against HEV in vitro, will probably need modifications to rule out undesired effects, in particular against the mitochondrial DNA polymerase.

### 3.2. NITD008 and GPC-N114

Recently, Netzler and colleagues tested a collection of 16 compounds with a reported inhibitory effect on the RNA-dependent RNA polymerase (RdRP) of different classes of viruses [30]. Both the class of NAs as well as non-nucleoside inhibitors (NNI) were considered in their study, covering compounds from late-preclinical stage to FDA-approved drugs. They identified two compounds, GPC-N114 and NITD008, inhibiting a GT1 derived subgenomic replicon. GPC-N114 had a half maximal effective concentration (EC50) of 1.07 µM and a therapeutic index (TI) of >93 (GPC-N114) whereas NITD008 had an EC50 0.03 µM and a TI >3333. GPC-N114 has been described as an NNI of the RdRP of multiple genera within the *Picornaviridae* family [31]. NITD008 has been reported to act as an antiviral on numerous viruses, amongst others HCV [32], West Nile Virus [33] or Zika [34]. NITD008 has shown toxicity in vivo [35]. So far, no clinical trials have been registered for both of these compounds.

### 3.3. Ciprofloxacin and IFN-λ

Nishiyama and colleagues used an FDA approved drug library for an in vitro screen against a GT3 strain containing a *Gaussia* luciferase reporter [36]. Ciprofloxacin (CPFX) and IFN-λ1-3 showed the best activity against the reporter genome. They also used an infection model, where cells infected with a full-length GT3 strain at a plateau phase were co-cultivated with naïve cells. All IFN-λ subtypes, but not CPFX, showed robust reduction of HEV RNA.

### 3.4. Sofosbuvir

Sofosbuvir is a nucleotide prodrug, which can be incorporated into HCV RNA by the NS5B RdRp acting as a chain terminator [37]. Its antiviral potential against HEV was demonstrated using GT3 HEV replicons in Huh7 and HepG2 cells. The IC_50_ values were in a low micromolar range, 1 to 2 orders of magnitude less potent than against HCV replicons [25]. Sofosbuvir showed an additive effect together with RBV. The inhibitory effect was confirmed by the same group in induced pluripotent stem-cell derived hepatocyte-like cells (iPSC-HLCs) for all four common GTs [38] and in cell culture for GT1 [30] as well as GT3 [36] by other groups. However, it was also described that both GT1 and GT3 replicons as well as full-length GT3 virus were not inhibited in HEK293T, U-87MG, and Huh7 cells [39]. Very recently, Li et al. reported that a GT5 strain was moderately inhibited at 200 µM by an isopropyl ester of Sofosbuvir, PSI 7977 [40].

Similar to the situation in vitro, the data on Sofosbuvir’s efficacy in vivo have been inconclusive. Three case studies stated that Sofosbuvir failed to clear HEV in patients when administered together with RBV [41,42,43], while three reported successful treatment together with RBV [44,45,46]. A multicenter phase II study addressed whether Sofosbuvir monotherapy is efficacious against HEV. A total of 400 mg Sofosbuvir was administered once daily over a course of 24 weeks to nine patients who had previously failed RBV therapy [47]. None of the patients cleared the virus, although it reduced HEV RNA levels by at least one order of magnitude in five out of nine patients during the study period and significantly reduced alanine aminotransferase (ALT) levels. With its antiviral efficacy being only moderate, it is probably not suited for use as monotherapy. However, it could further be investigated in combination with RBV.

## 4. Screening

Compound screening allows for the rapid testing of a large number of chemical substances and extracts with the aim of identifying new compounds. In the context of viral infections, important drugs identified with this method include Maraviroc and Etravirine [48], both of which target HIV as well as Daclatasvir, which targets HCV [49]. Prerequisite for compound screening is a suitable assay, reflecting the physiological conditions of infection. Importantly, the screening method has the benefit of not necessarily requiring profound knowledge about the viral lifecycle or specific targets. Assays using purified enzymes, subgenomic replicons or full virus already have successfully been used in HCV research.

### 4.1. Plant Ethanol Extracts

There have been two reports about the antiviral activity of ethanol extracts of plants against HEV. One extract was prepared from the plant *Lysimachia mauritiana* and showed activity against a pSHEV3-luc replicon in Huh7.5 cells, as well as while a full-length GT3 virus on RNA and protein level in A549 cells [50]. However, identification of the compound(s) responsible for the antiviral effect was not performed. The same group reported the antiviral effect of an ethanol extract of another plant, *Liriope platyphylla*, against HEV [51]. Both a pSHEV3-Luc-replicon as well as the 47832c full-length genome were inhibited by the extract in Huh7.5 cells. By performing activity-guided fractionation and multicolumn chromatography, Spicatoside A could be identified as the active compound, and its activity as a pure compound was demonstrated. No testing for toxicity, resistance induction or in vivo efficacy was conducted.

### 4.2. Zinc

Zinc is an essential micronutrient that has been reported to reduce replication of, amongst others, HIV [52] and Coronavirus [53] in high concentrations. In a study investigating the influence of different salts on HEV replication, it was demonstrated that Zinc salts inhibited replication of both GT1 and 3 replicons as well as a GT1 clinical isolate in pORF4-Huh7 cells [54]. The effect was likely due to an inhibition of the RdRP, as an inhibitory effect on the protein was observed in vitro. A mild effect could be observed at 10 µM, being strongest at 200 µM. It will be of interest to investigate if zinc supplementation will prove an effective strategy against HEV in patients. Zinc levels in plasma have been reported to be usually around 10–20 µM depending on the study, as described in a meta-analysis [55]. The authors also found that zinc supplementation increased plasma levels, with every doubling of the dose leading to a 9% increase [55]. These data imply that zinc levels are highly regulated, suggesting that effective plasma levels in patients might be at best challenging to reach. There has been no evaluation of zinc monotherapy against HEV in vivo so far. However, a case study [56] investigated the influence of intra-erythrocyte zinc levels on the outcome of RBV treatment. They showed that treatment successes could not be attributed to increased zinc levels. Although only comprising four patients in total, this suggests that it might not lead to viral clearance in immunocompromised individuals when combined with RBV.

### 4.3. 66E2

66E2 is part of a small molecule compound library belonging to the diversity set II of National Cancer Institute Developmental Therapeutic Program. It was initially identified as an inhibitor of the RdRP of HCV, inhibiting HCV 3a replicon with an EC_50_ of 2.5 µM [57]. The compound also inhibited HEV, both the replicon p6-Luc as well as full-length p6 in Huh7 or Huh7 S10-3 cells. Interestingly, it did not show inhibitory effect on purified RdRP for both HCV and HEV, suggesting that it does inhibit replication either by a affecting a host factor or that it does undergo modification within the host. No testing resistance induction or in vivo efficacy was performed.

## 5. Basic Research/Structure-Guided

**Figure 2:**
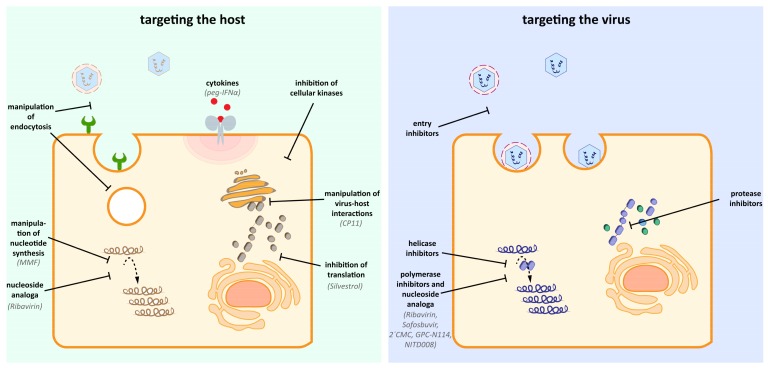
Potential host and viral targets of antiviral drugs. Antiviral therapy against HEV can rely on the inhibition and manipulation of host components, which are important for the HEV life cycle. Additionally, direct acting antiviral specifically can target viral enzymes (e.g., helicase, polymerase, protease) without affecting host components. Notably, the nucleoside analog Ribavrin is reported to exert antiviral effects by targeting both the virus and the host [19].

Different from screening approaches, target- or structure-guided development aims at identification of suitable targets for an intervention first. Prerequisite is that the function of a target is characterized. Both the virus itself and the host factors necessary for the viral life cycle can be targeted (Figure 2). Only one protein of HEV has been crystallized so far, which is a truncated version of the capsid protein pORF2 [58,59]. Although some conclusions regarding the involvement of certain domains in cell binding could be drawn, and heparan sulfates seem to be important for HEV attachment to the host cell surface [60], this knowledge has not led to the establishment of a therapy concept yet. The scarcity of antiviral candidates directly acting on the virus can be explained by the lack of knowledge about HEVs molecular virology.

Due to their small genomes, viruses depend on host factors for completion of their life cycle. This dependence on the host is a potential point to intervene. Host-acting antivirals have the potential to interfere with the distinct steps of the viral life cycle, from blocking the entry receptor over preventing the formation of the replication complex to hamper the viral maturation by inhibiting cellular proteins. A detailed functional analysis of the host factor and its role in the context of HEV infection is desirable and would help to optimize chemical intervention.

### 5.1. Hammerhead Ribozymes

A Hammerhead ribozyme is a small catalytically active RNA molecule, capable of cleavage or self-cleavage [61]. Hammerhead ribozymes have been designed to target, amongst others, severe acute respiratory syndrome coronavirus (SARS-CoV) [62] or HCV [63]. However, there has only been one report so far describing the use of this technique in vivo [64], which dates back already more than a decade. In 2003, it was reported that a hammerhead ribozyme could be designed to specifically cleave RNA of HEV GT1 in the 3’cis-acting element [65]. When expressed from a vector in HepG2 cells, they could decrease replication of a pSGI-HEV-3’Luc construct. Questions about delivery of the agent to target site in vivo in patients, as well as potential off-target effects, might remain unsolved, considering that there has been no follow-up study so far.

### 5.2. Peptide Conjugated Phosphorodiamidate Morpholino Oligomers

Peptide conjugated phosphorodiamidate morpholino oligomer (PPMO) are uncharged nucleic acid analogs containing a morpholine backbone connected by phosphorodiamidate linkage, rendering them resistant to many enzymes like nucleases, esterases, and proteases [66]. PPMOs bind RNA via Watson–Crick base pairing and interfere with viral translation by steric blockage. PPMOs complementary to sequences in Sar55 have been shown to inhibit replication of a pSK-E2 replicon in Huh7 S10-3 cells. They also reduced pORF2 levels in HepG2/C3 cells infected with Kernow C1 and Huh7 S10-3 cells infected with pSK-E2 [67]. So far, there has been no follow-up on this study.

### 5.3. MG132

MG132, an inhibitor of the proteasome with low nanomolar inhibitory constant [68] has been suggested as an antiviral against HEV. It reduced RNA and protein levels of a HEV GT1 replicon in Huh7 S10-3 cells [69]. However, when others reproduced the experiment in Huh7 cells, they also found reduced expression of several housekeeping genes as well as overall RNA levels [70]. It is therefore assumed that MG132 inhibitory effect on HEV is unspecific.

### 5.4. CP11

Some viruses can bind the cellular protein tumor susceptibility gene 101 (TSG101) to hijack the ESCRT machinery for egress, for instance, HIV-1 [71]. It was demonstrated that pORF3 of HEV can bind TSG101 via a PSAP motif in the viral protein and does colocalize with it in HEV-transfected cells [72]. Consequently, pORF3 is essential for viral egress, while having a neglectable effect on cell binding and replication [73]. The interaction with TSG101 is mediated via the PSAP motif in the pORF3 [74], the same motif that p6Gag of HIV-1 uses to engage with the cellular protein [75]. Cyclic peptides (CP) that had been developed to abrogate interaction of p6Gag and TSG101 and inhibited viral release of HIV Virus like particles (VLPs) [76] were tested for their activity against HEV [77]. One of the inhibitors, CP11, inhibited interaction of TSG101 and pORF3 both in a yeast-three hybrid screen as well as in a pulldown assay. Viral release of both GT1 and GT3 HEV from Huh7 pORF4 or Huh7 cells, respectively, was inhibited without showing significant toxicity. Inhibition of viral release by targeting pORF3/TSG101 interaction is an interesting mechanism for antiviral development, with CP11 providing a starting point for further compound development and characterization.

### 5.5. Inhibitors of Inosine-5′-Monophosphate Dehydrogenase

In general, targeting production of nucleotide synthesis to deplete or imbalance nucleotide pools is a strategy employed against several viruses [78,79]. The compounds RBV and mycophenolic acid (MPA), both of which target enzymes involved in nucleotide synthesis, are either already used as treatment against HEV or have been reported for their potential to inhibit the virus. As its ester MMF, MPA inhibits the Inosine-5′-monophosphate dehydrogenase (IMPDH), which is a pivotal enzyme in the purine nucleotide synthesis and is part of immunosuppressive regimens in organ transplant recipients. In a German study, a tendency was identified for its use in heart-transplant recipients being linked to clearance of HEV infection without development of chronicity [80]. In Huh7 cells, MPA inhibited replication of a p6-Luc replicon, an effect that could be reverted by supplementation of guanosine, suggesting inhibition of IMPDH as inhibitory mechanism [81]. In the same study, an additive effect with RBV was found. These results could not be confirmed in patients. A French study assessed the effect of immunosuppression by MMF on RBVs’ potential to lead to a sustained virological response (SVR) [82]. They did not find evidence for an additive effect of MMF on RBV treatment. Because of its immunosuppressive effect, MMF monotherapy to treat HEV infections seems unlikely. Given the lack of evidence for an additive effect with RBV, it will probably also not be used to treat HEV infections together with RBV.

Although MMF seems unsuited as a drug against HEV, targeting the IMPDH or enzymes involved in the purine synthesis pathway might still be a starting point for further studies. In a proof-of-concept study, Wang and colleagues [83] tested 23 custom designed inhibitors of the IMPDH. All inhibitors decreased replication of a p6-Luc replicon, emphasizing the potential of this target. Furthermore, inhibitors of pyrimidine synthase like Brequinar and Leflunomide inhibited the replicon. This is especially interesting, since both drugs have been tested in clinical trials and are therefore better characterized than newly developed inhibitors. Targeting nucleotide synthesis, especially of pyrimidines, might therefore be an interesting approach to tackle HEV, with Brequinar and Leflunomide already providing starting points for testing in vivo. It would be of interest to characterize these compounds better to evaluate their potential as HEV antiviral.

### 5.6. Silvestrol

Silvestrol is a natural compound belonging to the class of cyclopenta[b]benzofuran compounds, which are exclusively found in plants of the Aglaia genus [84]. Silvestrol has been shown to target the translation initiation factor 4A (eIF4A) to RNA, thereby preventing ribosome loading onto mRNA and blocking translation [85]. Originally described in the context of cancer treatment, silvestrol has been reported to inhibit several viruses in vitro [86,87,88]. Silvestrol reduced viral titers, the number of infected A549 cells, as well as viral protein levels in infected cells in vitro [89]. Another study confirmed the inhibitory effect of silvestrol on GT3 replicons in HepG2 cells with an IC_50_ of around 5 nM for GT3 HEV [90]. The inhibitory effects were consistent for different patient isolates covering the HEV GT1-4, which were evaluated in iPSC-HLCs. Silvestrol reduced viral titers in feces in xenograft mice infected with HEV GT1, therefore demonstrating its effectiveness in vivo. Importantly, silvestrol was effectively inhibiting a p6-Luc replicon harboring the G1634R mutant, which confers RBV resistance [91,92]. These data demonstrate that silvestrol might provide a therapy option for otherwise untreatable RBV resistant cases. Characterization of potential resistance barriers and a structure–activity relationship of the compound will be important next steps for further development of a promising candidate.

## 6. Vaccine

Vaccination may be an important strategy to reduce the global burden of the virus. In China, there is a vaccine licensed under the name Hecolin^®^. It consists of the amino acids 368–606 of the capsid E2 protein of HEV GT1, produced in *E. coli* [93]. Three doses at 0, 1, and 6 months were tested in healthy patients 16–65 years of age [94]. Long-term studies following the same cohort up to 54 months after vaccination showed an efficacy of 85.1 % in the intention-to-treat-analysis as well cross-protection against HEV GT4 [95]. There are currently several open questions about the vaccine, thereunder its efficacy and safety in risk groups, the cross-reactivity against other HEV GTs, and long-term protection after more than 54 months.

It is unknown whether Hecolin^®^ is safe for pregnant women and their fetuses, which are a population especially at risk of fatal outcomes of HEV GT1 infection. Results obtained from pregnant women that had unintendedly been enrolled by mistake in the Phase III study suggest that the vaccine might be safe for them [96]. However, because the study was not designed for that purpose, this finding is not a definitive conclusion. A study that does not exclude pregnant women is currently running in Bangladesh and expected to finish in 2020 (ClinicalTrials.gov Identifier: NCT02759991). It is also unknown if the vaccine is protective in the second risk group of immunosuppressed or immunocompromised patients. A French study investigated re-infections with HEV in organ transplant recipients seropositive for anti-HEV IgG before transplantation [97]. They showed that an IgG titer up to 6.2 WHO units/mL does not guarantee protection from a re-infection up to one year after transplantation. The data from the long-term study on Hecolin^®^ [95] indicate that the geometric mean of IgG titers 13 months after the first dose is already below that value in individuals seronegative at baseline. Notably, the IgG titers of individuals, which were anti-HEV IgG positive before vaccination, fell below the threshold of 6.2 WHO units/mL after 31 months. This raises the question whether chronic HEV infections in immunocompromised patients can be attacked with the current vaccine. There are also no data on safety and efficacy in the risk group of patients with chronic liver disease. It is unknown if the vaccine confers cross-protection against HEV GTs 2, 3, 7 or rat HEV, although the cross-reactivity of the vaccine against GT4 HEV is promising. The vaccine schedule requires six months; it would be to clarify if the vaccine can be used short-term to combat an outbreak. These limitations and uncertainties make research on antiviral therapy an important issue, regardless of the vaccine’s benefits.

## 7. Limitations, Needs, and Hopes for HEV Drug Development

**Figure 3:**
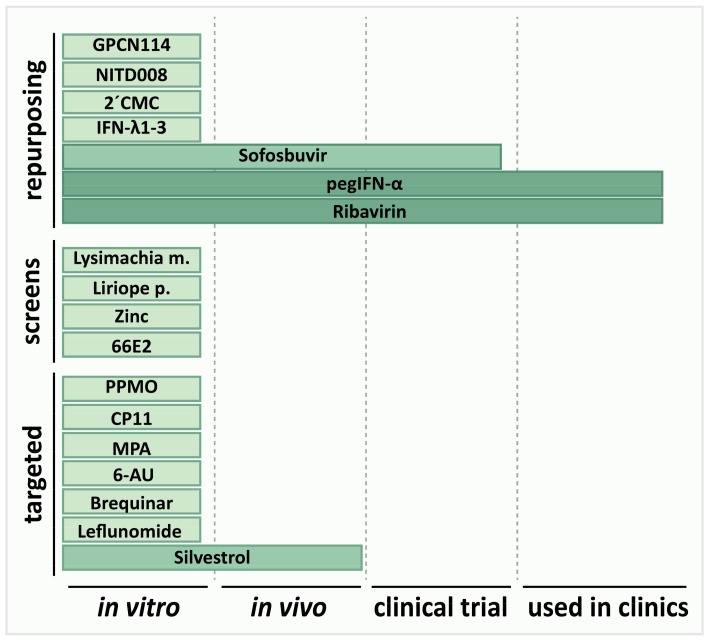
Overview of molecules/extracts with antiviral activity against HEV. The depicted molecules/extracts are classified according to the strategy that was used to identify them. So far, the antiviral activity against HEV of only four drugs (Sofosbuvir, pegIFN-α, Ribavirin and silvestrol) was approved in experimental settings beyond in vitro cell culture systems. Both drugs in the clinics are used off-label and therefore have not been tested in clinical trials against HEV.

The recently evolving attention on HEV also led to an increased focus on finding a satisfactory therapy. However, Sofosbuvir is the only candidate in clinical trials to date (Figure 3). Preliminary results indicate that it will not be a breakthrough in HEV therapy options. Apart from that, the nature of repurposed drugs might make them appealing to use for clinical trials. However, a 2-CMC derivative was discontinued due to toxic effects, while GPC-N114 or NITD008 never moved to clinical trials, which might indicate that they have adverse effects that could hinder development to an antiviral. The hits identified from other screens are either not well characterized yet, including toxicity, or are difficult to dose in vivo. Poor characterization is also an issue for several of the target-based compounds, while one is an immunosuppressant. Inhibition of pORF3 and TSG101 might be an interesting target for drug development, as demonstrated using CP11. Silvestrol is a promising candidate, since it is comparably well characterized and shows efficacy in vivo and an additive effect with RBV. Despite some challenges like scaling up its production, silvestrol is the most promising candidate at the moment. Although the vaccine can be of great use in reducing HEV burden, there are too many open questions, especially about their efficacy in risk groups and outbreak scenarios to solely rely on it to combat HEV.

An ultimate goal would be to discover specific agents only targeting the viral enzymes as, for instance, the HEV protease or polymerase. These so-called direct acting antivirals (DAAs) are highly specific and were a breakthrough in HCV therapy with high cure rates and enhanced efficacy. Being virus-specific, they might also be much more suited for the use in immunocompromised patients and especially pregnant patients, for whom there is no therapy yet. Of note, none of the drug candidates presented in this paper has an approval for use in pregnant women. Integral for the discovery of DAA was in the case of HCV and will be in the case of HEV the crystallization of the respective enzyme structures. This will enable structure-guided design of potent inhibitors by fitting the compound and the viral enzyme to complementary surfaces. As mentioned above, the structure of pORF2 has been determined, but without further implications for drug development yet. Regarding the polyprotein from ORF1, it is still debated whether it is cleaved into fragments upon translation or not. Some studies argue that it is not cleaved [98,99,100,101], while others report cleavage into several fragments [102,103]. Recently, a study was published suggesting that the proteases factor X and Thrombin cleave the polyprotein and that silencing of these reduced pSK-HEV2-Luc replication [104]. The assignment of functional domains to the pORF1 polyprotein was done in 1992, according to similarity to proteins in related viruses that had known functions [105]. Of these seven domains, the functionality could be confirmed with biochemical assays for four: Methyltransferase [106], papain-like cysteine protease [107], Helicase [108], and RdRP [109]. The exact function of the other domains as well as the exact borders of each functional domain are not known to date. Until it is not completely understood how the polyprotein is processed and how this does affect the structure and enzymatic activity of the functional domains, studies to identify antivirals based on structural insights seem at best unlikely. To date, the de novo development of candidate structures into a potential licensed drug is only a future perspective, underlined by the fact that none of the anti-HEV candidates has been designed based on a structure of an HEV protein.

There are also only a few host factors known; therefore, the identification of novel host factors will be another cornerstone in combating HEV. All -omics approaches to decipher the altered cellular environment during infection, as well as functional studies using cDNA, shRNA and siRNA libraries to overexpress or silence host proteins, are powerful and successful tools to discover novel host factors. This will both give new starting point for drug discovery as well as potentially refine in vitro and in vivo models.
